# Influenza

**Published:** 1891-06-27

**Authors:** 


### The Practitioner's Mirror and Retrospect.

[*The Editor will be glad to receive offers of co-operation and contributions from members of the profession. All letters should be addressed to* The Editor, The Lodge, Porchester Square, London, W.]

### MEDICINE.

### INFLUENZA.

The medical art suffers much reproach at the present time because it can tell so little of the cause or nature of this ubiquitous disease, and the failure to thus explain or classify is undoubtedly stranger since it is no new or rare enemy, but one which has attacked humanity in its own violent fashion at short intervals from probably the earliest ages. The history of the recorded epidemics is marvellously complete for centuries. Hirsch tabulates the outbreaks from the year 1173, and adds that there were many others before them. He fills twelve pages with titles of the works in which the epidemics of influenza for these 700 years alone are recorded. Thomson gives elaborate accounts of 20 outbreaks in England alone in the last three centuries and a-half, and yet we seem little nearer to an explanation of its causes than in the days when men first began to record its ravages. Every country and every climate in the world is subject to it, yet it appears to find a permanent home nowhere as a constant or endemic resident, but to disappear from the face of the globe for a series of years. Hitherto no disease has shown a completely analogous course. The ordinary infective fevers which, in the infancy of science, might have been thought to follow a similar inscrutable career, have been shown never to arise spontaneously. All outbreaks of a given fever either spring through successive infections from an original common ancestor, or have some natal country where their germs reproduce themselves age after age in the earth or water as well as in the human body. We have no trace of either source for influenza, except, indeed, some slight evidence for its endemic character in sub-arctic countries which lacks confirmation. Yet its symptoms follow precisely the type of the other infective fevers, and preserve a remarkable uniformity and individuality in successive epidemics. Every analogy is against our regarding it as a violent outbreak of ordinary catarrh induced by specially inclement seasons. It must either be the product of some germ which occasionally migrates or attains pathological qualities, or of one which lies dormant in the bodies of infected persons for 20 or 40 years. In the latter supposition the present outbreak represents the periodical ague fit of those who suffered in 1847, together with the first attacks of those they infect. It is far more probable that the germ has some undiscovered endemic source, or that it exists usually in a harmless form, as is said to be the case with that of certain varieties of pneumonia.

The disease has frequently broken out in ships at sea, when not a trace of it had occurred at their previous ports. Others it has actually met at sea on coming towards countries where it was prevalent. This was noted in certain war vessels coming from the Mediterranean to the English coast in 1847, and in many others. Clearly, the contagion, if there be one, is capable of floating vast distances through the air. Yet it is wholly independent of the wind. Thus, the disease crept during 1837 from south to north in England against a north-east wind, and from north to south in Iceland in 1834 during a steady west wind.

Nor does it spread gradually from place to place, but by leaps and bounds; places that have been missed may escape altogether or be attacked many weeks later, and distant places, such as London and Cape Town, may be smitten at the same moment. Last year, and on several other occasions, it travelled gradually from the east to the west of Europe, but Gluge holds that the earliest epidemics followed an opposite course.

The eccentricities of cholera and typhoid have been shown to depend chiefly on infection through the water supply, but such explanation is impossible here. Sometimes it picks out the troops or prisoners from a town, and at others passes over them, and no race of men have immunity. Neither the climate, the dampness of the soil, the season of the year, the temperature, nor state of the weather, have much influence on the outbreaks. They rage under the burning heat of the Phillipines, and in the cold and mist of the Faroe Islands, equally well in dry or wet seasons.

Now it seems perfectly clear that while the infection is spread differently to that in most diseases, an infection of some kind does exist. Thus, frequently, an outbreak has occurred on the arrival of ships from abroad, as the dwellers in Iceland and the Faroe Isles have noticed. It is curious also that outbreaks of yellow fever have very often followed the arrival of ships, while attendants on the sick generally escape.

Nothnagel and Leyden think that influenza spreads only through the air. Henoch considers it contagious, while Hirsch denies this. In favour of Henoch's view, it is noticeable that in a district of Bristol last year, in houses where any cases occurred, very nearly a half of the inhabitants fell ill, whereas the proportion of the whole population was far less. Again, a vessel from St. Nazaire to Vera Cruz, took up a passenger at Santander, who came from Madrid where the disease was rife. In four days influenza broke out, and 150 out of 430 fell ill.

On the other hand, the collective investigation undertaken in 1837, showed that rarely, if ever, was the disease communicated from one person to another. If with Squire we hold that infective germs are of two kinds, those which multiply only in the human body and which are propagated by direct infection, and against which a previous attack is protective; and on the other side those which multiply external to the body, and which are only indirectly infectious, then influenza would, like malarial fevers, fall under the second class, but its appearance only in epidemics at such long intervals, and the extraordinary rapidity with which it makes its way over the world separate it, as we have said, from all others. One peculiarity is suggestive—it often attacks only the natives and thoroughly acclimatised members of a given population while new-comers escape, even though they came in the ships after whose advent the disease broke out. The inference is probable that the new-comers have been protected by recent exposure, and that the germ of influenza is more widely present in a mild form than we should otherwise suppose, though the protection may only last a short time. In this way we may explain the difficulty of pointing out the cause of the disease, the germ to which it is owing being common enough in an innocent form. Klebs thought that he had discovered this in certain flagellate protozoa found in the plasma or corpuscles of the blood during the febrile stage, but no cultivations were made. Gruber found nothing in the blood, but a diplococcus like Fränkel's in the sputum. Ribbert thinks that the streptococcus pyogenes is invariably present, and is the actual cause of influenza; but Besser showed that it was common in healthy men at least during the epidemic. Seifert, in 1884, found a micrococcus in the sputa of influenza patients and of no others; while Koch declared his belief that we have here to do with a class of micro-organisms quite different from bacteria, and which at present we are unable to cultivate outside the body. Of these he instanced the protozoa found in malaria, and which have quite lately attracted attention through Golgi's photographs. They appear to be universally present in ague, and by their segmentation give rise to the paroxysms. Now, the form of these protozoa changes so rapidly, that only by very numerous observations can we demonstrate, as Golgi claims to have done, that the various appearances belong to different stages of the same amœba. In malaria we have to do with a disease always present, while in influenza the difficulties of research are enormously increased by its absence after an epidemic has run its course. Fischel has also found an organism in the blood during the febrile stage which he succeeded in cultivating, and by which he produced symptoms in horses and dogs not unlike those of influenza. In horses an epidemic has often occurred co-incidently with that among human beings, and Hirsch regards it as related; but the name has been applied to two other equine complaints, and we must not forget the difficulties caused by a hasty identification of tuberculosis in fowls with that in man. In view of the numerous epidemics in the past, we cannot be too cautious in referring influenza to such a cause as the Chinese floods. The infection is certainly not solely, if at all, transmitted from one patient to another. It apparently produces a ptomaine which has marked effects on the nervous system and the heart, and it is probable that we shall be able to isolate this poison before we can demonstrate its cause.

